# Microgravity triggers ferroptosis and accelerates senescence in the MG-63 cell model of osteoblastic cells

**DOI:** 10.1038/s41526-023-00339-3

**Published:** 2023-12-16

**Authors:** Nancy Garbacki, Jérôme Willems, Thibaut Neutelings, Charles Lambert, Christophe Deroanne, Astrid Adrian, Markus Franz, Matthias Maurer, Philippe De Gieter, Betty Nusgens, Alain Colige

**Affiliations:** 1 Laboratory of Connective Tissues Biology, GIGA-Cancer, University of Liège, 4000 Liège, Belgium; 2 Airbus Defence and Space, GmbH, 88090 Immenstaad, Germany; 3 European Space Agency (ESA), European Astronaut Centre (EAC), 51147 Cologne, Germany; 4 European Space Agency (ESA), 2201, AZ, Noordwijk, The Netherlands

**Keywords:** Cell biology, Molecular biology

## Abstract

In space, cells sustain strong modifications of their mechanical environment. Mechanosensitive molecules at the cell membrane regulate mechanotransduction pathways that induce adaptive responses through the regulation of gene expression, post-translational modifications, protein interactions or intracellular trafficking, among others. In the current study, human osteoblastic cells were cultured on the ISS in microgravity and at 1 g in a centrifuge, as onboard controls. RNAseq analyses showed that microgravity inhibits cell proliferation and DNA repair, stimulates inflammatory pathways and induces ferroptosis and senescence, two pathways related to ageing. Morphological hallmarks of senescence, such as reduced nuclear size and changes in chromatin architecture, proliferation marker distribution, tubulin acetylation and lysosomal transport were identified by immunofluorescence microscopy, reinforcing the hypothesis of induction of cell senescence in microgravity during space flight. These processes could be attributed, at least in part, to the regulation of YAP1 and its downstream effectors NUPR1 and CKAP2L.

## Introduction

Cells in the body experience different types of stimuli, which coordinately regulate their structure, function and fate. Besides regulations operated by soluble factors (hormones, growth factors, metabolites, etc.) and cell–cell interactions through specific receptors, mechanical forces are recognized as potent cell regulators^1–3^. In this context, an extensive characterization of the molecular mechanisms by which cells sense and react to mechanical loading or relaxation should help refine our current understanding of cell physiology and pathology.

On Earth, life is exposed to persistent gravitational force. As a result, its role in physiology is hard to investigate and has long been underestimated. Since the advent of long-duration spaceflights in recent decades, it has become obvious that astronauts exposed to microgravity develop many health problems, the most notably affected tissues being the musculoskeletal system^4,5^. In this context, efforts have been made to characterize the mechanisms involved. Indeed, such knowledge could lead to the identification of potential therapeutic targets to prevent space-related health alterations, but also to approaches to treat a range of pathological conditions common on Earth, whether related to aging (such as osteoporosis or skin atrophy), to an altered response to mechanical stimuli or to the induction of abnormal mechanical stress (such as fibrosis, keloids, restenosis after angioplasty).

To decipher the mechanisms potentially involved in bone mass loss in astronauts at the molecular level, a large number of experiments using bone cells in culture have been carried out over the last few decades. According to a recent meta-analysis^6^, different types of bone cells (osteoblasts, osteoclasts and mesenchymal stem cells) have been studied. Most of these experiments, however, used cells of animal origin (mouse, rat and fish) and were carried out on Earth using simulated microgravity models (2D clinostats, random positioning machine, etc.) that provide continuous changes in orientation with respect to the gravity vector, thus averaging out towards zero the gravity vector felt by the cells.

Although these models have proved to be useful, they do not provide true microgravity and space environment, including radiations. Methods to generate more genuine microgravity, including sounding rockets, parabolic flights and free fall from drop tower, also have limitations due to their short duration, which prevents investigating long-term regulations. Furthermore, most of these experiments used analytical techniques such as RT-qPCR or microarray, restricting the number of studied genes or reducing the sensitivity of gene expression quantification^7^. Despite their cost and limited availability, experiments conducted in space remain unrivaled for studying the influence of microgravity on cellular phenotype. The aim of our experiment, which uses the MG-63 osteoblastic cells as an experimental model, was to understand how cells perceive and respond to the loss of gravity in a real space environment, using both morphological analyses and high-throughput RNA sequencing (RNAseq). This project was selected by NASA/ESA following a call for research proposals and approved for a scientific mission conducted in the BioLab incubator on the International Space Station (ISS).

## Results

### Experimental design

Cell biology experiments to be carried out in microgravity (µg) in the ISS are facing several constraints. The first one is related to the delay between the preparation of the cell cultures in the laboratory, the integration of the culture devices in the rocket, the travel to the ISS and the storage until the availability of crew members to start the experiment. In addition, launches are often postponed (from several hours to many days), which can have a deep impact on cell phenotype and viability. Finally, during the period between cell preparation and the start of the experiment, it is impossible to prevent vibrations, period of hypergravity and temperature variations, which are potential bias influencing cell behavior and interpretation of data^8–10^.

In order to mitigate these undesirable side effects and to benefit from the most relevant controls, we decided to send cells frozen as monolayers in a cryoprotective medium, and kept at –80 °C until the start of the experiment on the ISS, avoiding the above-mentioned problems. The entire experimental framework was first extensively studied and improved on Earth by testing many different parameters concerning, notably, the composition of the cryoprotective medium and cell freezing parameters, culture conditions and timing, sample fixation and long-term storage procedures, adaptation of our experimental conditions to the specificity of the hardware, and the reliability of the post-experimental analyses. Ultimately, we identified optimal conditions in which frozen monolayers can be stored at –80 °C for up to 6 months without significant alteration of cell viability and without compromising post-experimental analysis. Immediately after thawing by the crew, all the cultures were maintained at 37 °C for 38 h in a centrifuge running at 1 g to allow cells to recover from the thawing stress at the Earth gravity level and to be similarly pre-conditioned before starting the active phase of the experiment comparing cells at 1 g and µg.

**Fig. 1:**
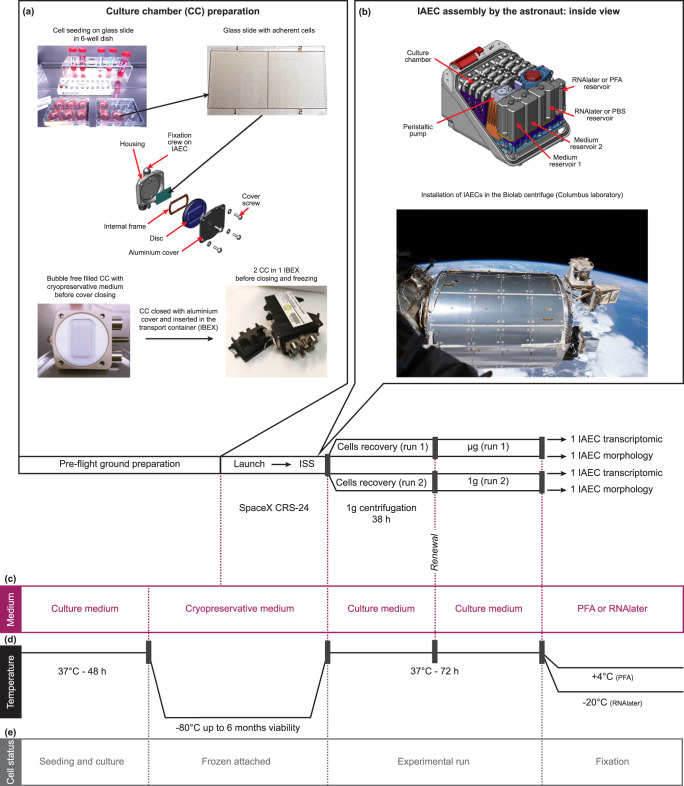
Mission profile of SpaceX CRS-24 and experimental concept. **a** MG-63 cells were seeded on glass slides and cultured for 40 h, before being integrated in culture chambers (CC). The culture medium was then replaced by cryopreservative medium, and CC were inserted in protective transport containers (IBEX), progressively frozen and stored at –80 °C until start of the experiment onboard. The experiment consisted in two sequential runs, the first one containing the µg samples and the second one the 1 g flight controls. The CC were removed from the –80 °C freezer 90 min before starting the run in order to allow progressive thawing. **b** They were then installed in the Integrated Advanced Experimental Container (IAEC) together with the medium and fixative reservoirs. The IAECs were integrated into a BioLab centrifuge and the cryopreservative medium was immediately replaced by culture medium. The entire procedure is described in the Methods section. The BioLab incubator was warmed to 37 °C and the centrifugation at 1 g was initiated for 38 h to allow cell recovery. At the end of the 1 g recovery phase, the medium was renewed and the centrifuge containing the cultures to be exposed to microgravity was stopped (run 1) and cultured for a further 34 h. For run 2, the centrifuge kept running at 1 g for 72 h. The runs were terminated by fixation either with RNAlater (for transcriptomic analyses) or with PFA followed by PBS washing (for morphological evaluations). The status regarding **c** culture medium or fixative, **d** temperature variations and **e** cells are illustrated. Image credit: “Columbus over Earth”, ESA–L. Parmitano at https://www.esa.int/ESA_Multimedia/Images/2020/01/Columbus_over_Earth (CC BY-SA 3.0 IGO).

During the first run, the centrifuge containing the cultures to be exposed to microgravity was stopped after the 38 h recovery period, while, during the second run, the centrifuge containing the control samples kept running at 1 g after the recovery period. At the end of the runs, cells were fixed with PFA or RNAlater and stored at appropriate temperatures until downloading and shipment to the laboratory. A scheme of the entire process is provided (Fig. 1), and additional details are given in the Methods section.

### Transcriptomic analysis

Transcriptomic analyses were performed by RNAseq on cells cultured in µg and at 1 g in the ISS (an informative transcriptomic comparison between the 1 g on board and a ground experiment is provided in Supplementary Note 1 and Supplementary Data 1). A total of 10,092 genes were found to be expressed with a quantifiable base mean count (>10) but not significantly altered by microgravity (|log2 fold change| < 0.33). This list includes hnRNPs (a large family of RNA-binding proteins contributing to mRNA transcription, stabilization and translation), mitochondrial proteins (MT-CO1, MT-CO2, MRPLs), and glycolytic enzymes (ENO1, GAPDH). Panther database and GO enrichment analyses^11,12^ were performed using this set of unaltered genes. These analyses led to the identification of 298 biological processes and functions (*p* value < 0.05) (Supplementary Data 2) related to “Gene transcription and translation”, “Spliceosome”, “rRNA processing”, “RNA methylation”, “Regulation of signal transduction”, amongst others. These data clearly show that the overall cell functioning was not excessively affected in our experimental conditions, allowing searching for specific regulations elicited by microgravity.

**Fig. 2:**
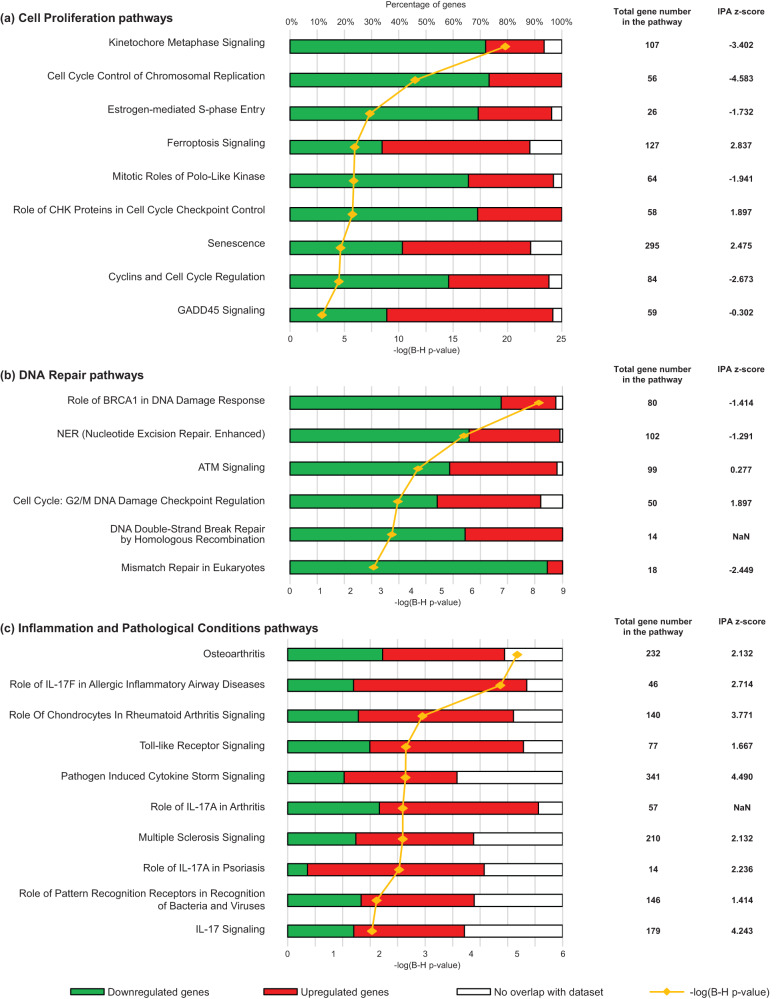
Main cell functions and pathways with the highest correlation to genes that are differentially expressed in µg as compared to 1 g flight control. Canonical pathways identified by IPA were grouped regarding their cell function: **a** cell proliferation, **b** DNA repair and **c** Inflammation and pathological conditions. The different individual pathways were classified according to their –log(B–H *p* value), a corrected *p* value based on the Benjamini–Hochberg method, which is a powerful tool to decrease the false discovery rate (yellow line and symbols). For each individual pathway, the percentage of genes that are downregulated, upregulated and not significantly regulated in regard to our analysis cut-offs are illustrated by color code (green, red or white, respectively). The total gene number in the pathway and the *z*-score calculated by IPA are also indicated, with positive and negative *z*-score values indicative of stimulation or inhibition, respectively. Also see Supplementary Data 4–7 describing the entire canonical pathways analysis.

Using a more stringent cut-off (|log2 fold change| > 1, base mean counts >100, adjusted *p* value < 0.001), 998 genes were identified as significantly differentially expressed in µg conditions (508 downregulated and 490 upregulated) as compared to 1 g (Supplementary Data 3). Twenty-four regulatory pathways were found to be highly significantly modified in microgravity (Benjamini–Hochberg (B–H) *p* value < 0.001), while 16 additional pathways had a B–H *p* value < 0.01 (Supplementary Data 4). Data corresponding to pathways with significant –log(B–H *p* values) and for which an associated *z*-score was calculated (Fig. 2) were further clustered into three more general cellular pathways.

The “cell proliferation” pathway (Fig. 2a) contains a majority of downregulated genes (Supplementary Data 5), suggesting an inhibition of cell proliferation in microgravity. In line with this, “Ferroptosis” (a particular cell death pathway dependent on iron) and “GADD45 signaling” (notably leading to growth arrest) pathways are both upregulated.

“DNA repair” (“Nucleotide excision repair”, “Homologous recombination” and “Mismatch repair”) forms a second category of pathways composed of a majority of downregulated genes (Fig. 2b and Supplementary Data 6). Alterations in these pathways are possibly involved in the cell cycle arrest and the reduced cell proliferation, notably through the activation (*z*-score = 1.9) of the “G2/M DNA Damage Checkpoint Regulation” pathway.

The third large category of cellular functions altered in microgravity contains pathways with a high proportion of upregulated genes (Supplementary Data 7) which are mostly related to inflammation, cell stress and pathological conditions (Fig. 2c).

Considering these three categories together, it is clear that the cell phenotype is affected by microgravity. However, it is worth noting that, except for ferroptosis, other pathways related to cell death, such as apoptosis or necrosis, are not altered, showing that microgravity elicits specific regulation or adaptation, and not a more general dysregulation of cell phenotype.

In addition to these general considerations on transcriptomic data, a brief description of the above-mentioned pathways is given in more detail below to provide an overview of the potential impact of microgravity on cell phenotype.

### Cell proliferation pathways

The kinetochore metaphase signaling is the most significantly altered pathway in microgravity. Kinetochores allow physical interactions between chromosomal centromere and microtubules during mitosis. They ensure the fidelity of chromosome segregation during mitosis and recent data indicate that their dynamics is regulated by physical tensions and stress, with impacts on mitosis^13^. In this pathway, the vast majority of genes affected by microgravity are repressed by a factor around 2–3, including the CEPN proteins of the inner plate and many of the proteins forming the outer plate (SKA1, SKA2, SKA3, NDC80, NUF2, SPC24, SPC25, KNL1, ZWINT, NSL1), likely leading to alteration of chromosome segregation during mitosis (Supplementary Data 5).

The expression of genes implicated in the cell cycle control of chromosomal replication during S-phase is significantly impacted by microgravity across all the steps required to assemble the replicative complex before the elongation of newly synthesized DNA. Origin recognition complexes *ORC1* and *ORC6*, genes of the mini-chromosome maintenance complex (*MCM2* to *MCM 10*) and checkpoint regulators (*CDC6*, *CDC45*, *CDC7*) and replication genes (*POLA1*, *POLA2*, *POLE*, *PRIM1*, *TOP2A*) are downregulated with a minimum of 2-fold change (Supplementary Data 5).

Among the 127 genes involved in ferroptosis signaling, the IPA software identified 112 genes as being differently expressed in microgravity, predicting an activation (*z*-score = 2.8) of this pathway (Fig. 2a). A list of the 23 most significantly differentially expressed genes (|log2 fold change| > 1, base mean counts >100, adjusted *p* value < 0.001) is provided in Table 1. Altogether, these genes encompassed most of the aspects related to iron biology, such as iron storage (the ferritin chains, *FTH1* and *FTL*), transmembrane transporters (*SLC39A8*, *SLC39A14*, *SLC11A2*, *SLC7A11* and *SLC3A2*), oxidation of Fe^2+^ to Fe^3+^ (ceruloplasmin), transcription factors (*NFE2L2*, *BACH1*, *STAT3*, *ATF4*, *TP53*) and genes implicated in lipid peroxidation (*SAT1*, *ACSL4*, *ABCA1*). Such an extended impact on a specific pathway identifies ferroptosis as a potential key node for understanding the deleterious effects of microgravity.

**Table 1:** Ferroptosis pathway genes modulated by microgravity.

Ensembl ID	Gene symbol	Gene name	Log2 fold change	Fold change	*p* value
ENSG00000165029	ABCA1	ATP binding cassette subfamily A member 1	2.805	6.989	2.33E-44
ENSG00000068366	ACSL4	Acyl-CoA synthetase long-chain family member 4	1.979	3.942	2.41E-55
ENSG00000167772	ANGPTL4	Angiopoietin like 4	1.983	3.953	6.80E-11
ENSG00000168374	ARF4	ADP ribosylation factor 4	1.185	2.274	2.96E-13
ENSG00000128272	ATF4	Activating transcription factor 4	1.079	2.113	3.29E-09
ENSG00000124762	CDKN1A	Cyclin-dependent kinase inhibitor 1A	1.149	2.218	1.66E-21
ENSG00000128965	CHAC1	ChaC glutathione specific gamma-glutamylcyclotransferase 1	1.943	3.845	7.22E-13
ENSG00000172071	EIF2AK3	Eukaryotic translation initiation factor 2 alpha kinase 3	1.220	2.329	2.27E-10
ENSG00000144554	FANCD2	FA complementation group D2	–1.326	0.399	1.89E-24
ENSG00000167996	FTH1	Ferritin heavy chain 1	2.654	6.294	1.03E-63
ENSG00000087086	FTL	Ferritin light chain	1.532	2.892	2.52E-25
ENSG00000001084	GCLC	Glutamate-cysteine ligase catalytic subunit	1.030	2.042	4.00E-10
ENSG00000184270	H2AC21	H2A clustered histone 21	–1.278	0.412	1.08E-09
ENSG00000105968	H2AZ2	H2A.Z variant histone 2	–1.012	0.496	3.60E-34
ENSG00000278588	H2BC10	H2B clustered histone 10	–1.256	0.419	1.48E-10
ENSG00000274641	H2BC17	H2B clustered histone 17	–1.592	0.332	2.90E-28
ENSG00000162409	PRKAA2	Protein kinase AMP-activated catalytic subunit alpha 2	1.104	2.149	5.47E-14
ENSG00000080839	RBL1	RB transcriptional corepressor like 1	–1.104	0.465	8.84E-15
ENSG00000130066	SAT1	Spermidinespermine N1-acetyltransferase 1	2.722	6.598	2.71E-38
ENSG00000138821	SLC39A8	Solute carrier family 39 member 8	1.304	2.469	6.24E-06
ENSG00000104635	SLC39A14	Solute carrier family 39 member 14	1.731	3.320	3.24E-32
ENSG00000151012	SLC7A11	Solute carrier family 7 member 11	1.282	2.432	1.21E-09
ENSG00000161011	SQSTM1	Sequestosome 1	1.519	2.866	9.02E-31

List of genes modulated by a |log2 fold change| > 1 (fold change >2 or <0.5). Analysis cut-off: base mean count >100, adj *p* value <0.001. Expression *p* values were calculated based on the Benjamini–Hochberg procedure.

The expression of 261 genes out of the 295 genes forming the senescence pathway was identified as modified in µg (Fig. 2a), including 37 genes characterized by highly significant statistical values (|log2 fold change| > 1, base mean counts >100, adjusted *p* value < 0.001) (Table 2). Even if gene expression heterogeneity can be observed during senescence^14^, the modulation of some of them is considered as a hallmark of senescence. Amongst them, the overexpression of the cell cycle regulator *CDKN1A* (p21) and of pro-inflammatory cytokines (*IL1A*, *IL6*), and the repression of the expression of lamin B1 (*LMNB1*) and E2F transcription factor 2 (*E2F2*) were also found in the µg samples.

**Table 2:** Senescence pathway genes modulated by microgravity.

Ensembl ID	Gene symbol	Gene name	Log2 fold change	Fold change	*p* value
ENSG00000162772	ATF3	Activating transcription factor 3	2.605	6.084	3.35E-32
ENSG00000134107	BHLHE40	Basic helix-loop-helix family member e40	1.835	3.568	6.50E-26
ENSG00000149260	CAPN5	Calpain 5	–1.035	0.488	1.01E-16
ENSG00000173894	CBX2	Chromobox 2	–1.003	0.499	3.76E-14
ENSG00000134057	CCNB1	Cyclin B1	–1.438	0.369	4.20E-26
ENSG00000157456	CCNB2	Cyclin B2	–1.347	0.393	1.21E-24
ENSG00000175305	CCNE2	Cyclin E2	–1.422	0.373	1.45E-30
ENSG00000164045	CDC25A	Cell division cycle 25A	–1.136	0.455	3.49E-11
ENSG00000158402	CDC25C	Cell division cycle 25C	–1.383	0.383	7.74E-18
ENSG00000170312	CDK1	Cyclin-dependent kinase 1	–1.283	0.411	3.29E-23
ENSG00000124762	CDKN1A	Cyclin-dependent kinase inhibitor 1A	1.149	2.218	1.66E-21
ENSG00000172216	CEBPB	CCAAT enhancer binding protein beta	1.593	3.017	2.11E-20
ENSG00000169429	CXCL8	C-X-C motif chemokine ligand 8	3.415	10.666	5.07E-23
ENSG00000101412	E2F1	E2F transcription factor 1	–1.641	0.321	1.58E-21
ENSG00000007968	E2F2	E2F transcription factor 2	–2.074	0.238	1.54E-36
ENSG00000133740	E2F5	E2F transcription factor 5	1.232	2.349	6.77E-13
ENSG00000165891	E2F7	E2F transcription factor 7	–1.667	0.315	6.67E-29
ENSG00000129173	E2F8	E2F transcription factor 8	–1.754	0.296	6.04E-13
ENSG00000106462	EZH2	Enhancer of zeste 2 polycomb repressive complex 2 subunit	–1.396	0.380	7.83E-28
ENSG00000099860	GADD45B	Growth arrest and DNA damage-inducible beta	1.461	2.753	1.22E-10
ENSG00000105856	HBP1	HMG-box transcription factor 1	1.443	2.719	2.85E-13
ENSG00000136244	IL6	Interleukin 6	5.317	39.864	1.20E-84
ENSG00000115008	IL1A	Interleukin 1 alpha	2.978	7.879	6.93E-23
ENSG00000113368	LMNB1	Lamin B1	–1.580	0.334	3.36E-40
ENSG00000196611	MMP1	Matrix metallopeptidase 1	1.341	2.533	1.46E-05
ENSG00000149968	MMP3	Matrix metallopeptidase 3	2.763	6.788	2.83E-10
ENSG00000004799	PDK4	Pyruvate dehydrogenase kinase 4	3.724	13.214	1.62E-36
ENSG00000119403	PHF19	PHD finger protein 19	–1.379	0.384	1.04E-35
ENSG00000080839	RBL1	RB transcriptional corepressor like 1	–1.104	0.465	8.84E-15
ENSG00000162302	RPS6KA4	Ribosomal protein S6 kinase A4	–1.113	0.462	2.08E-18
ENSG00000137834	SMAD6	SMAD family member 6	–1.722	0.303	2.07E-21
ENSG00000101665	SMAD7	SMAD family member 7	1.042	2.059	1.13E-11
ENSG00000112096	SOD2	Superoxide dismutase 2	1.787	3.451	7.63E-06
ENSG00000161011	SQSTM1	Sequestosome 1	1.519	2.866	9.02E-31
ENSG00000119699	TGFB3	Transforming growth factor beta 3	1.655	3.149	1.90E-04
ENSG00000137462	TLR2	Toll like receptor 2	1.378	2.599	4.74E-06
ENSG00000185650	ZFP36L1	ZFP36 ring finger protein like 1	1.081	2.116	1.58E-31

List of genes modulated by a |log2 fold change| > 1 (fold change >2 or <0.5). Analysis cut-off: base mean count >100, adj *p* value <0.001. Expression *p* values were calculated based on the Benjamini–Hochberg procedure.

Cyclins and cyclin-dependent kinases (CDKs) are regulatory proteins necessary for proper progression through the transition phases of the cell cycle, as well as for the regulation of transcription and DNA repair. Their expression is particularly affected by microgravity with potential impacts on the four phases of the cell cycle. Cyclins (*A2, B1, B2, E2*), *CDK1*, *CDK2* and *CDC25* phosphatase (regulating key transitions between cell cycle phases) are downregulated, whereas cyclin-dependent kinases inhibitors *1A* and *1B* are upregulated (Supplementary Data 5), leading to a potential inhibitory activity on the G1, S, G2 phases transitions and on mitosis.

### DNA repair pathways

Pathways involved in the “DNA damage response” are presented in Fig. 2b. BRCA1 is involved in DNA repair and cell cycle checkpoint regulation. It is part of the “Double strand break repair by homologous recombination” and the “Role of BRCA1 in DNA Damage Response” pathways. *BRCA1*, *BRCA2*, *BARD1*, *RAD1* and *FANCD2* are significantly downregulated, potentially resulting in defective organization of the BRCA1 and FANC/BRCA complexes, which could lead to an alteration of DNA repair (Supplementary Data 6). Upon activation, CHK1 phosphorylates a variety of substrate proteins, resulting in the activation of DNA damage checkpoints, cell cycle arrest, and DNA repair or cell death. Within the E2F family of transcription factors E2F1 and E2F2 are considered as typical activators of DNA repair processes, while E2F5 is an inhibitor^15^. Finally, p53 (TP53) is a positive regulator of CDKN1A, which is a potent inhibitor of cell cycle. Altogether, these observations suggest that microgravity could potentially result in reduced DNA repair capacity. It must be stressed however that many of the involved regulations depend on complex and dynamic phosphorylation/dephosphorylation processes that could not be evaluated in this study.

### Inflammation and pathological conditions pathways

Several interleukins and chemokines are considered as markers of various types of cellular stress following exposure to radiation, ROS, inflammation, mechanical stretching and relaxation, or endoplasmic reticulum dysregulation. Many of them are strongly upregulated in microgravity environment, explaining why several pathways related to inflammation (“osteoarthritis”, “rheumatoid arthritis”, several “IL-17 related” pathways) were identified (Fig. 2c and Supplementary Data 7).

*IL6* is the most upregulated gene, with a ~40-fold change in microgravity as compared to the 1 g condition. *IL1A*, *IL1B* and *IL33* are also strongly induced, as well as *CXCL1*, *3*, *5*, *6* and *8*. PTGS2 (also known as COX2) is the inducible form of cyclooxygenase responsible for prostaglandins synthesis. Its strong overexpression in microgravity, around 18-fold change, is an additional relevant indicator of cellular stress. Several transcription factors being part of this pathway are also overexpressed. The transcription factor activator protein 1 (AP1) is a heterodimer predominantly made of FOS and JUN, which are both overexpressed in microgravity. Besides being a strong inducer of many cytokines involved in inflammation, AP1 also promotes the expression of a wide spectrum of other proteins and proteolytic enzymes such as the extracellular matrix-degrading enzymes MMP1, MMP2, MMP3, which are all three overexpressed in our microgravity dataset.

### RT-qPCR validation of RNAseq analysis

In order to validate our RNAseq results before assessing in greater detail the biological implications of the regulations observed in microgravity, RT-qPCR was carried out on selected transcripts chosen on the basis of their relevance. These analyses revealed a close correlation (*r* = 0.99 and *p* < 0.0001, Spearman test) between RNAseq and RT-qPCR data, confirming the reliability of RNAseq quantifications (see Supplementary Note 2).

### Upstream regulators

Based on the entire set of transcriptomic data, we used the IPA “Upstream Regulator and Causal Networks” analytical tool to identify key regulators that have the highest probability of being implicated in the microgravity-induced regulations, either directly or indirectly. In these analyses, positive or negative *z*-score values are indicative of, respectively, activation or inhibition of the predicted regulators.

A total of 218 potential upstream regulators were identified (overlap *p* value < 0.001, |*z*-score| > 2), among which NUPR1 (*z*-score = 10.5; 134 modulated genes) and CKAP2L (*z*-score = –6.2; 39 modulated genes) have the highest and the lowest scores, respectively (Supplementary Data 8). NUPR1 and CKAP2L are both regulated by the Yes-associated protein (YAP1), a transcription factor of the Hippo pathway, involved in mechanosignaling^16,17^. YAP1 is also predicted (*z*-score = –3.0) to be an upstream regulator of the microgravity-induced transcriptional modifications. However, as its activity is determined by phosphorylation at different sites depending on a complex network of regulators, the absence of microgravity-induced modification of its expression (fold change = 1.05) was not surprising. The expression of *LMNB1* (lamin B1, fold change = 0.33), *KIF23* (a kinesin, fold change = 0.39) and *PLK1* (a kinase implicated in cell cycle regulation, fold change = 0.38) are known to be dependent on NUPR1, CKAP2L or YAP1/Hippo pathways. Of additional interest, two other lamins, *LMNA* and *LMNB2*, were also downregulated (fold changes = 0.54 and 0.42, respectively).

### Morphological analysis by fluorescence staining

**Fig. 3:**
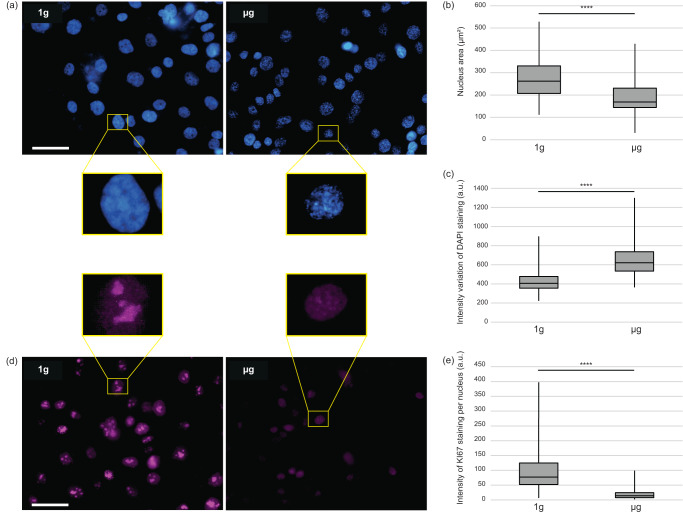
Comparison of the nuclear morphology and the KI67 staining levels in cells at 1 g flight control and µg. **a** DAPI nucleus staining (blue) in 1 g control and µg cells. **b** Quantification of nuclear apparent surface in 1 g control (*n* = 170) and µg cells (*n* = 190), **c** showing a reduction of the size of the nuclei in µg. Fluorescence intensity variations within individual nucleus (variations between the background and bright spots inside the nucleus) was measured in 1 g control cells (*n* = 164) and in cells in µg (*n* = 146). A higher heterogeneity of DAPI staining was observed in cells in µg. **d** KI67 (violet) staining in 1 g control and µg cells. A strong reduction of KI67 staining was observed in µg, which is illustrated on higher magnification views of representative nuclei and confirmed by **e** quantification of nucleus-associated violet staining (*n* = 102 in 1 g, *n* = 135 in µg). Box plots indicate the median, with the top and bottom borders of the boxes representing the 75th and 25th percentiles, respectively, and the whiskers above and below the boxes indicating the maximal and minimal values. Statistical analyses were performed with the Mann–Whitney *U* test (****p value < 0.0001). Scale bars are 50 µm. a.u. arbitrary units.

The morphology of nuclei was evaluated (Fig. 3a, b). No nuclear fragmentation is observed in microgravity, indicating that apoptosis was not induced, in line with transcriptomic data. A significant reduction (*p* < 0.0001) of the median area of the nuclei is observed in cells exposed to microgravity (166 µm^2^) as compared to cells at 1 g (259 µm^2^), potentially reflecting repression of the cell cycling, again in agreement with the transcriptomic data. In control cells at 1 g, the staining of the nucleus appears quite uniform, while, instead, numerous spots of more intense DAPI fluorescence were observed in microgravity (Fig. 3c). These differences indicate a microgravity-induced formation of facultative heterochromatin, a process known to be associated with cell stress.

Ki67 is a nuclear antigen used as a marker of active cell proliferation. In cultures at 1 g, the vast majority of cell nuclei are KI67 positive (Fig. 3d), some further showing larger and strongly stained spots typical to late G1 phase. In microgravity, the staining, when present, is much fainter and diffuse (Fig. 3e). Although the abundance of KI67 is known to be regulated by an active degradation process, it is worth noting that our transcriptomic analyses show a 3-fold reduction of its expression in microgravity.

**Fig. 4:**
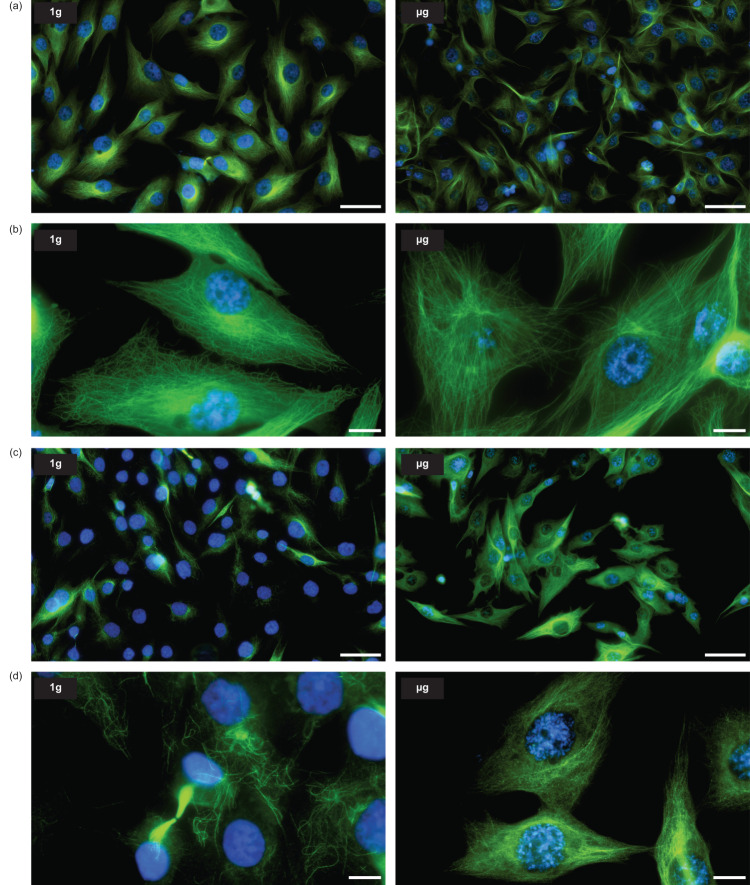
α-Tubulin and acetyl-α-tubulin architecture in 1 g flight control and µg cells. **a** Large field views of α-tubulin staining (green) and nuclei (blue) in 1 g and µg cells. **b** Higher magnifications show the organization of the microtubules in each gravity condition: an entangled wavy fibrils pattern in 1 g cells versus a straighter structural organization in µg cells. **c**, **d** Different field views of acetyl-α-tubulin staining (green) and nuclei (blue) in 1 g and µg cells. The staining is generally limited to a few microtubules in 1 g cell. Conversely, all the cells in µg are characterized by intense staining showing the presence of acetyl-α-tubulin organized in microtubules throughout their entire cytoplasm. Scale bars are 50 µm (**a** and **c**) and 10 µm (**b** and **d**).

Tubulins are the building blocks of microtubule filaments, which are key structures for several biological processes as mitosis and intracellular transport, among others. In 1 g condition (Fig. 4a, b, left panels), the α-tubulin staining shows a typical intertwined wavy network throughout the cytoplasm, while the microtubules in microgravity have a straighter, bundle or star-shaped orientation (Fig. 4a, b, right panels). The acetylated form of tubulin is reported to be present in long-lived stable microtubules such as found in centrioles and primary cilia. It participates in various cell functions including cell polarity, cell migration or vesicle transport. For cells maintained at 1 g, the acetyl-α-tubulin staining is faint, and present on a limited number of microtubules (Fig. 4c, d, left panels). Strikingly, the staining is much more intense for cells in microgravity (Fig. 4c, d, right panels), indicating that a large proportion of tubulin is acetylated likely affecting the function of microtubules.

**Fig. 5:**
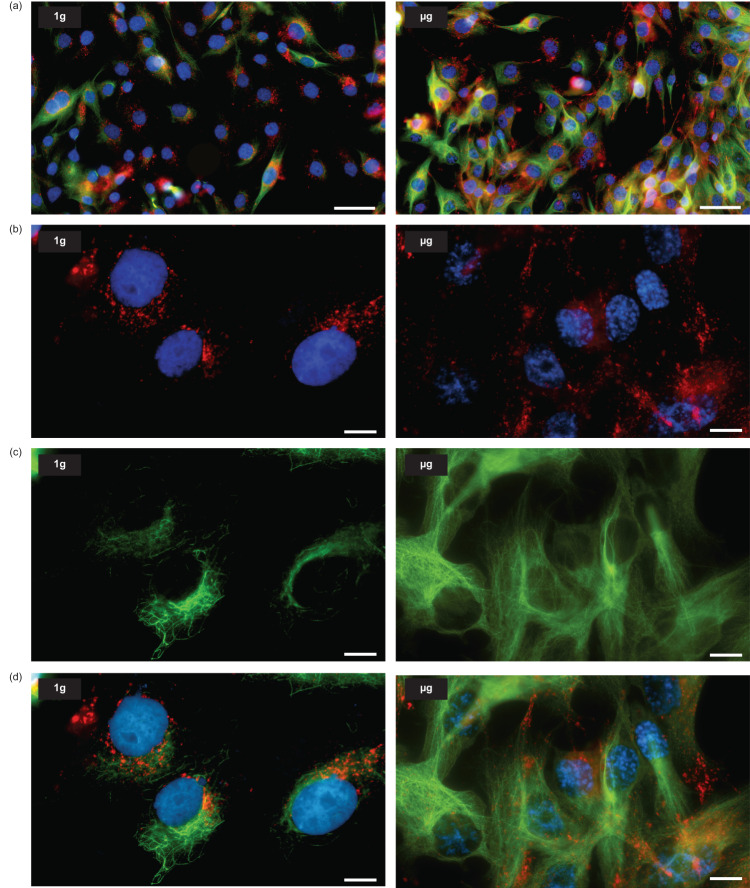
Effect of microgravity on lysosomes spatial distribution. **a** Large field views of 1 g flight control and µg cells after staining to visualize lysosomes (LAMP2, in red), nuclei (blue) and acetyl-α-tubulin (green). **b** Higher magnification of lysosomes (red) and nuclei (blue) and **c** of acetyl-α-tubulin (green) in representative 1 g and µg cells. Merged images from (**b**) and (**c**) are provided in (**d**). Lysosomes in 1 g control cells tend to clump close to the nucleus in regions enriched in acetyl-α-tubulin. Instead, they are scattered throughout the cytoplasm for cells cultured in µg. Scale bars are 50 µm (**a**) and 10 µm (**b**-**d**).

The localization of the lysosomes directly depends on microtubule-mediated trafficking and tubulin acetylation. It was evaluated by co-immunofluorescence staining (Fig. 5a) of LAMP2 (marker of lysosomes, Fig. 5b) and acetylated tubulin (Fig. 5c). In the 1 g flight condition, lysosomes are mostly present around the nucleus (at the microtubule minus-ends) whereas, in the µg cells, bright puncta of LAMP2 staining were dispersed throughout the cytoplasm with some accumulations at cellular extensions (at the microtubule plus-ends) (Fig. 5d).

**Fig. 6:**
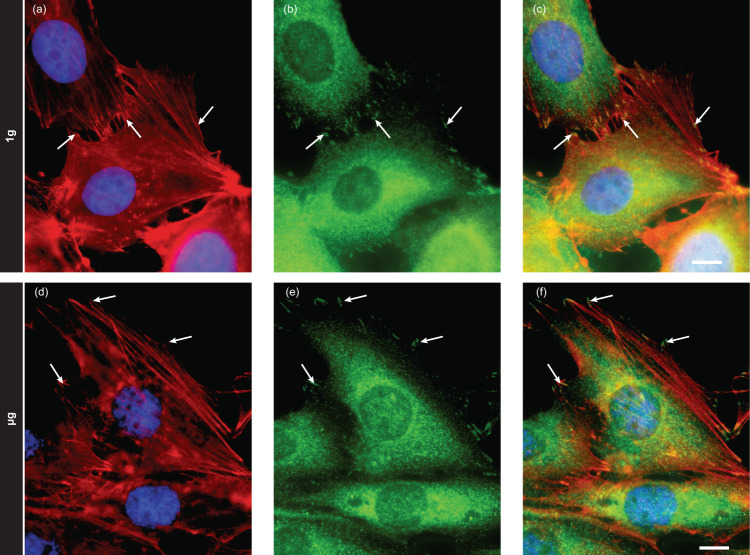
Fibrillar actin and pFAK staining in 1 g flight control and µg cells. **a**–**c** 1 g flight control cells and **d**–**f** cells in µg were stained for **a**, **d** fibrillar actin (red) and nucleus (blue) and for **b**, **e** pFAK (green). Merged channels are in **c**, **f**. The white arrows show some focal adhesions identified as green pFAK positive spots linked to actin fibers. Scale bars are 10 µm.

A recognized pathway of mechano-sensing involves the physical continuum linking the extracellular matrix to transmembrane receptors (such as integrins) to focal adhesions (developing at the intracytoplasmic domain of integrins), to the actin cytoskeleton and to the nucleus. The dynamic formation and disassembly of actin filaments, by linking focal adhesions to other cell structures, critically regulates a wide range of cellular processes, including cell migration, cell division, and gene expression. Two key actors of this critical pathway were evaluated: the actin cytoskeleton and the focal adhesions (identified by detecting the phosphorylated form of the Focal Adhesion Kinase (FAK), also known as PTK2). A similar staining of fibrillar actin and phospho-FAK was observed at 1 g (Fig. 6a–c) and in microgravity (Fig. 6d–f), which suggests that these factors are not involved in the observed microgravity-induced changes.

## Discussion

Loss of gravity experienced during space flight has profound effects on human physiology, including muscle atrophy, endocrine disorders, reduction in bone density and immune dysfunction. Whether these alterations result from the impact of weightlessness on the entire body or tissues or from regulatory mechanisms occurring at the cellular level remains much less documented. This is mainly due to the difficulties encountered in carrying out cellular experiments in space under conditions that allow precisely discriminating specific effects of microgravity from additional parameters, such as cosmic radiations and cell stress occurring between their preparation on Earth and the start of the experiment in space (temperature variations, vibrations and hypergravity during the launch, starvation, etc.). A way to avoid these confounding effects would be to compare cells in microgravity to control cells cultured in reconstituted 1 g gravity onboard in the same space environment. Our experimental study was designed accordingly. First, we designed and extensively validated protocols allowing the shipping and storage of cell cultures in the ISS as frozen monolayers, avoiding pre-experimental stresses. Based upon previous investigations, cultures were then thawed and allowed to recover for 38 h at 37 °C in a 1 g centrifuge, just before the start of the experiment. Although a previous pilot assay was conducted with a device designed to defrost cryopreserved cell cultures in the ISS^18^, to the best of our knowledge, our experiment is the first to successfully report biological data by using deep-frozen monolayers of adherent cells. Secondly, and most importantly, control cells were also cultured in the ISS, in the same conditions as cells in microgravity, but in a centrifuge running at 1 g for the total duration of the experiment, making them the most relevant controls. Finally, we combined transcriptomic analysis with morphological observations to obtain a comprehensive overview of microgravity-induced modifications.

The general morphology of cells was not altered in microgravity as compared to cells centrifuged at 1 g. Similarly, a large majority of genes were not differentially expressed. These were critical criteria to assess the reliability of our cell model.

In microgravity, KI67 staining was much less intense and the size of the nuclei was significantly reduced as previously reported for another osteoblastic cell line^19^. Both observations could indicate a reduction in cell proliferation. Apoptosis is probably not involved since none of its typical nuclear modifications was observed. Conversely, the presence of facultative heterochromatin, which is a marker of a senescence-associated proliferation arrest^20^, is noticeably increased in microgravity. In agreement with these morphological observations, transcriptomic analyses identified several repressed pathways in microgravity directly related to cell proliferation.

The organization of the cytoskeleton can affect both the nuclear morphology and cell proliferation. Previous data about alteration of the actin cytoskeleton in microgravity are quite confusing, with some reports showing its disorganization^21–24^, while some others describe increased F-actin and stress fiber formation^25,26^. In our study, we did not detect any significant change in the actin cytoskeleton, neither in the associated focal adhesions, which is in agreement with data reported by Rösner et al.^27^. One hypothesis to explain these discrepancies is that perturbations in the actin cytoskeleton organization would occur shortly after exposure to microgravity, followed by a rapid adaptation to the environment and restoration of a fully functional actin network^28^.

Microtubules represent another key component of the cytoskeleton. They are involved in cell polarity, cell shape and intracellular transport. They are also critically required during mitosis, which depends on several regulatory pathways identified from transcriptomic data, such as “Kinetochore metaphase signaling”, “Cell cycle control of chromosomal replication” or “Cyclins and cell cycle progression”. Correlations between their organization and mechanical forces applied on cells have been established. For instance, compressive forces tend to reduce any preferential orientation of the microtubules at the benefit of a more intermingled structure^29–31^ while cells grown on soft matrices, at low mechanical stress, show a straighter and less wavy orientation^32^. The latter type of architecture was observed in our cells in microgravity. The structure, dynamics and functions of the microtubules are further fine-tuned by post-translational modifications^33,34^. Tubulin acetylation participates in cell mechano-sensing by mediating focal adhesion dynamics^35^ and by increasing cytoskeletal stiffness and cellular viscoelastic resistance^36^. It regulates the microtubules functions in several cellular activities including cell polarity, cell migration and trafficking^37,38^. As compared to the 1 g control, we identified here a massive increase of tubulin acetylation in microgravity, affecting almost the entire microtubule network in every cell. In order to evaluate if it could affect some cell functions, the cytoplasmic distribution of lysosomes, a process depending on microtubules, was tracked. Impaired lysosomal function in the space flight environment was previously reported^39^. Lysosomes can be found as a rather immobile pool located close to the nucleus around the microtubule-organizing center in addition to a highly dynamic pool more homogeneously distributed, with some accumulation close to the cell periphery^40^. At 1 g, the large majority of lysosomes were found around the nucleus, partly co-localizing with sparse acetylated tubulin. Strikingly, they were much more dispersed in microgravity, even reaching the tips of some cell extensions, showing some correlations between the pattern of acetylated tubulin and lysosome distribution.

Cellular redistribution of lysosomes has been previously associated with a secretory phenotype related to senescence^41–43^. Several features induced in microgravity, such as alterations in tubulin structure and acetylation, chromatin condensation and repression of cell proliferation, are additional markers of cellular senescence^44–46^. Finally, excessive DNA damage possibly resulting from inhibition of the DNA repair pathways are also involved in senescence. The hypothesis of increased senescence in microgravity is further substantiated by our transcriptomic analyses that pointed the modulation of many senescence pathway-associated processes, such as, amongst others, the dramatic upregulation of pro-inflammatory cytokines and chemokines and of enzymes involved in extracellular matrix remodeling, which are features of the senescence-associated secretory phenotype (SASP) activation^47^. Downregulations of factors required for mitosis were also observed. Multiple triggers induce senescence, of which “DNA damage” and modifications of the mechanical properties of the environment, are the most relevant to our study. Beneficial and detrimental effects of senescence have been reported, depending on cell types and physiological or pathological conditions^48^. Our experimental setting does not allow deciphering if the signs of senescence identified here are part of a beneficial transient adaptation process or indicative of longer term deleterious modifications such as those leading to bone resorption for astronauts.

Ferroptosis refers to a specific type of programmed cell death where dysregulated iron metabolism increases the Fe(II) in the cytosol, resulting in the accumulation of lipid peroxides^49^. It has been shown that ferroptosis is pro-inflammatory and is associated with various human diseases, such as kidney injury, nervous system dysfunctions, blood-related pathologies or cancer^50^. Among the microgravity-altered genes implicated in the iron regulation, some encoding for major actors in the pathway were dramatically upregulated: the transferrin receptor (*TFRC*) necessary for cellular iron uptake, the ferritin complex (*FTH1* and *FTL*) that acts as a major Fe(II) intracellular storage to protect cell against reactive oxygen species (ROS), the pro-ferroptotic regulator *ACSL4* and the transferrin-independent Fe(II) transporters SLC39A8 and SLC39A14. Moreover, the overexpression of the transcription factor *ATF4* and of its downstream target *CHAC1* might lead to the degradation of glutathione, a major intracellular antioxidant^51^. Although the upstream cause cannot be identified here, our data therefore suggest that microgravity could lead to alterations of the iron transport and homeostasis, and be one of the cause of ROS production observed during space flight missions^52^.

Data regarding DNA damage induced in microgravity in space are conflicting, notably because, in most previous models, comparisons were made between cells cultured in space and control cells on Earth^53^. In these conditions, the alterations resulting from cosmic radiations or microgravity are difficult to distinguish from each other^54^. In our experiment, the samples in microgravity and the 1 g controls were all cultured in the ISS, therefore similarly exposed to radiations and, therefore, allowed investigating specifically the effect of microgravity. Transcriptomic analyses do not allow to directly track DNA damage, but can provide general insight into the activation or inhibition of key mechanisms involved in DNA repair. By comparing gene expression in microgravity versus 1 g in space, we identified a global repression of different DNA repair pathways which might potentially be of concern in long-duration human space explorations.

Transcriptomic analyses were used to identify which upstream regulators would have the highest probability of being involved. NUPR1 and CKAP2L were the two factors the most positively and negatively correlated with changes observed in microgravity. NUPR1 (Nuclear protein 1) is a transcriptional factor acting in response to multiple internal (DNA damage, ER stress, oxidative stress, inflammation, and metabolic stress) and external stress signals^55,56^. It is involved in a wide range of biological functions, including regulation of DNA damage and repair, cell cycle, autophagy, and cell death^57–68^. The structure and affinity of NUPR1 for its interactors are regulated by its phosphorylation by kinases, such as PKA^61^, which suggests an indirect activation by microgravity. This activation might arise from the increased intracellular ROS caused by the activation of the ferroptosis pathway^55^. CKAP2L (Cytoskeleton Associated Protein 2 Like) is a mitotic spindle protein^69^, but not much is known about its biology and functions, except that it is potentially implicated in some cancers and that mutations affecting its gene cause the Filippi syndrome^69^. As for NUPR1, CKAP2L is not expected to be a direct “sensor” of microgravity.

**Fig. 7:**
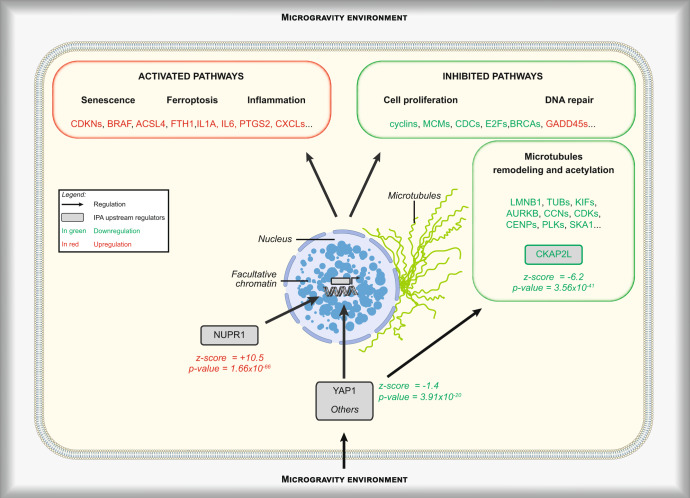
Illustration summarizing the regulations induced in microgravity as observed in our cell model. The “Cell proliferation” and “DNA repair” pathways are clearly inhibited as judged by the regulation of expression of some key genes. Conversely, “Senescence”, “Ferroptosis” and “Inflammation”, three pathways with partial overlaps, are strongly upregulated. Modifications in the structure of the microtubule network are another regulation induced in microgravity. As a way to identify the molecular mechanisms underlying these regulations, the transcriptomic datasets were further analyzed, using the “Ingenuity Pathway Analysis” software (IPA), in order to identify potential key upstream regulatory pathways. This analysis identified the YAP1 pathway as the most likely to be involved in the response to microgravity, by directly participating in the regulation of the expression of numerous modulated genes. This pathway is also known to be critically involved in mechano-sensing and mechanotransduction signaling and to be capable of regulating the other two likely upstream regulators, NUPR1 and CKAP2L. NUPR1 is a transcription regulator that converts stress signals into a program of gene expression that empowers cells with resistance to the stress induced by a change in their microenvironment, while CKAP2L is a microtubule-associated protein that is therefore implicated in cell cycle progression. The use of an inhibitor of the YAP1 pathway (verteporfin) in studies carried out on Earth reproduced some, but not all, of the regulations observed in microgravity (see Supplementary Note 3). This suggests that YAP1, although clearly involved, especially in the regulation of the activated pathways, is not the only regulator of microgravity effects, and that other mechanisms have yet to be discovered. These could affect NUPR1, CKAP2L or, more directly, the microtubule network. Chromatin remodeling (presence of facultative chromatin) and changes in the microtubule network (which may be regulated by CKAP2L) are also observed in microgravity. Interestingly, these features are also considered markers of senescence, making senescence the most central element of microgravity-induced changes.

The Hippo signaling pathway, and its effectors YAP1 and TAZ, is a key mechanism allowing cells to sense and respond to mechanical stimuli. The regulation of YAP1/TAZ activity depends mainly on their degradation and their nuclear localization, and, therefore, their gene expression was not expected to be modified in microgravity. Comparison of our data with previously published analyses supports the idea that microgravity might reduce YAP1 transcriptional activity. Barravecchia et al.^70^ observed a reduced nuclear YAP1 immunostaining signal in flight cells compared to the bright signal obtained on Earth. Liu et al.^16^ described a list of 45 genes upregulated in fibroblasts due to YAP1 activation or in response to increased matrix stiffness, of which 37 were downregulated by at least two-folds in our cells in microgravity, a situation corresponding to mechanical unloading. Most interestingly, the expression of both *NUPR1* and *CKAP2L* can be regulated by YAP1^16,17^, suggesting that the Hippo pathway might be central to our observed microgravity-induced modifications (Fig. 7).

Our experiment was designed to identify the effects, at the cellular level, of true microgravity in space, getting rid of other confounding effects such as exposure to cosmic radiations or to highly specific culture conditions. This study identified the “Ferroptosis” and “Senescence” pathways as being activated in microgravity, which could be directly related to downregulation of the DNA repair process. Although it could not be directly confirmed during the present study, our data suggest that the Hippo signaling pathway could be a hub in these cellular adaptations, which would be in line with many reports identifying it as a regulator in response to cell mechanical microenvironment on Earth^71–73^. Of direct interest, a recent publication suggests that this pathway could also be implicated in microgravity-induced liver dysfunction^74^. Due to its critical importance in diverse mechanisms and functions, including embryogenesis, tissue repair, cardiovascular diseases and cancer, the Hippo signaling pathway could be at the center of most of the previously observed cellular alterations observed in microgravity and should be investigated in depth^75^. Drugs targeting different actors in this pathway are becoming available. Their use in models relevant to microgravity and mechanical signaling, such as cultures in matrices of different stiffness, could provide crucial advances in the comprehension of health problems affecting astronauts during long-term missions and should contribute to a better understanding of age-related disorders.

## Methods

The CYTOSKELETON experiment was performed in the BioLab incubator of the European laboratory module Columbus on the ISS. It was selected by NASA/ESA following a Research Opportunities call (ESA/NASA ILSRA-2001-074) and approved for a mission on the ISS by ESA’s Advisory Group. Further to ESA, the project received the financial support of Prodex and the Belgian Science Policy Office.

### Experiment development, optimization and validation

We developed, tested, optimized, validated and established all experimental procedures, margins, timelines, operational and logistics procedures. These processes took place in the Laboratory of Connective Tissues Biology, University of Liège, Belgium and were conclusively validated by Experiment Sequence Test (EST) performed in Cologne, Germany, with the operational support of scientists from DLR (German Aerospace Center) and BIOTESC (Biotechnology Space Support Center, Luzern). The experiment was designed to mitigate the effects of transport, vibrations and hypergravity episodes during launch by sending cells deep frozen as attached monolayers. Moreover, deep frozen adherent cells, unlike actively growing cells, can support potential delay of the launch or in the ISS schedule as we demonstrated they can stand ready to use for up to 6 months when stored at –80 °C.

### Choice of experimental cell model and its potential limitations

Bone remodeling is one of the most widely described alterations in microgravity, explaining why we decided to use cells of osteoblastic origin. The development of the equipment and procedures, as well as the entire experiment to be carried out in the ISS, is a very long process requiring in-depth validation over several years. As a result, primary human osteoblasts are unsuitable because of their limited lifespan and phenotypic heterogeneity from batch to batch. The MG-63 osteoblastic cell line is widely characterized and has an unlimited lifespan. These cells respond to specific external stimuli (such as 1,25(OH)2 D3) like bone cells, and their integrin subunit profile is similar to that of primary osteoblasts, suggesting similar interactions with their environment. They are therefore considered a relevant model of pre-osteoblasts and are widely used in preclinical bone tissue engineering models^76–78^. Furthermore, they have been used in several cell biology space experiments^79,80^.

However, this model has certain intrinsic limitations. It cannot be ruled out that MG-63 cells may have an increased or reduced sensitivity to microgravity compared with normal osteoblasts. However, this limitation also applies to “normal” primary cell types (osteoblasts, fibroblasts, endothelial cells, immune cells) due to their phenotypic heterogeneity. Moreover, in many respects, cancer cells are more resistant to stress and external signals than their “normal” counterparts. Several preliminary tests were carried out to define the experimental parameters to be used in the study, including cultures in g-vector-modifying devices (clinostat and centrifuge). As compared to other cell types (fibroblasts, mesenchymal stem cells, other bone cancer cells), no specific sensitivity of MG-63 was detected, and they were therefore considered suitable for being investigated in microgravity.

### MG-63 cell culture and cryopreservation of attached cells

MG-63 osteosarcoma cells were cultured in DMEM (Gibco #31600-083) supplemented with MEM non-essential amino acids (1%, Biowest #MS00B3100H), penicillin 10 kU/streptomycin 10 kU (1%, Lonza #DE17-602E), amphotericin B (0.2%, Gibco #15290-018), gentamycin (0.1%, Gibco #15750-037), HEPES (12.5 mM, Gibco #15630-080), fetal bovine serum (10%, Gibco #10270-106), pH 7.4. Culture glass slides (24 × 14 × 1 mm) were successively washed in boiling water, 1 M HCl, distilled water, ether and 70% ethanol, before being dried until use. Cells were seeded as monolayers at a density calculated to ensure confluence after 40 h of culture at 37 °C.

Cryopreservation medium was made of 1:1 dilution of DMEM described above, supplemented with HEPES (final concentration 25 mM) and N-acetyl-L-cysteine (30 mM, Sigma #A9165), with cryoprotective medium (Lonza, #12-132A, DMSO final concentration 7.5%). Seeded glass slides were inserted in culture chambers (Fig. 1) filled with cryoprotective medium and directly inserted in protective transport containers (IBEX). Each IBEX was wrapped in plastic bubble foil, placed in a foam box and frozen at –80 °C. This packaging was experimentally validated to ensure an optimal cooling rate to avoid formation of deleterious intracellular ice. The highest post-thawing viability of adherent cells was obtained with a cooling rate of 1.6 °C min^−1^ from RT (20 °C) to –20 °C, followed by a cooling rate of 0.8 °C min^−1^ from –20 to –80 °C.

To control the viability of cell cultures prepared for flight, spare culture chambers units were thawed in the laboratory and submitted to a complete experimental run in a ground model of Integrated Advanced Experimental Container (IAEC), according to the protocol to be used on the ISS.

The frozen IBEX was transported to KSC at –80 °C and maintained at this temperature during launch, transfer and storage in the ISS until start of the experiment.

### Space flight and ground control experiment hardware

Briefly, the main hardware consisted of (1) culture chambers containing MG-63 cells, (2) the IAEC equipped with a peristaltic pump and waste bags, (3) culture medium reservoirs, (4) fixative reservoirs. Prior to the mission, the Liquid Management Systems (LMS) of the IAECs were prepared. The flow rate of each pump was set to obtain 2.5 ml min^−1^ (for cryoprotective medium replacement) or 3 ml min^−1^ (for all other liquid transfers). The system was sterilized with a solution of antibiotics (penicillin/streptomycin 200 U ml^−1^ each, amphotericin B 1 µg ml^−1^, gentamycin 100 µg ml^−1^ for 3 h at RT), and then rinsed with 70% ethanol (for 3 h at RT), before being vacuum emptied and dried for 5 days at 37 °C. The waste bags were also carefully vacuumed. The reservoirs were filled bubble-free in the laboratory. The culture medium reservoirs were packed in IBEX and frozen at –80 °C for transport. The fixative reservoirs filled with RNAlater (Invitrogen #AM7024 95% in sterile distilled water, for the transcriptomic analyses), or with paraformaldehyde (PFA) or with sterile PBS (for morphological analyses) were packed in IBEX, stored and then transported at a minimum of 16 °C to avoid salt crystallization (RNAlater reservoirs) or at –20 °C (PFA and PBS reservoirs). The filling of LMS tubing, with antibiotics diluted in sterile PBS, was finalized at the Space Station Processing Facility at the Kennedy Space Center.

### Launch

The CYTOSKELETON experiment was launched with SpaceX CRS-24 from Kennedy Space Center, LC-39A on 21st December 2021. IBEX with MG-63 cells, chemicals and IAEC were transported at the defined temperatures and stored accordingly after docking to the ISS until start of the experiment.

### Experiment sequence on the ISS

IBEX transport containers with culture medium reservoirs were transferred to the BioLab glovebox the day before starting the experiment for complete thawing. IBEX with cells culture chambers were transferred from the –80 °C freezer into the glovebox 1.5 h before the beginning of the experiment in order to allow progressive thawing. Cell culture chambers, medium and fixative reservoirs were assembled on the IAECs in the Life Sciences Glovebox (LSG) by the German astronaut Matthias Maurer. Two IAEC were dedicated to morphological analysis (PFA fixation) and two IAEC to transcriptomic analyses (RNAlater fixation). Two fully assembled IAEC (one for transcriptomic and one for morphological analyses) were installed in the BioLab rotor per run. The command schedule, prepared by BIOTESC and validated during the EST, consisted of (1) a first medium exchange to replace thawed cryopreservative solution by culture medium, first in the PFA IAEC and then in the RNAlater IAEC, (2) progressive increase of incubator temperature to 37 °C and start of the rotor to provide 1 g for 38 h (recovery period for both µg and 1 g runs), (3) second medium exchange, (4) incubation at 37 °C during 34 h under microgravity (run 1: rotor stopped) or at 1 g (run 2: rotor running), (5) sample fixation for transcriptomic (RNAlater) or morphological (PFA) analyses. A first run was performed in microgravity (µg condition) followed by the 1 g experiment (1 g flight control).

At the end of the experiment, the IAECs were uninstalled and transferred for storage at –80 °C in Polar 4 (samples in RNAlater for transcriptomic analysis) or at +4 °C in the BioLab TCU (PFA fixed samples and stored in PBS for morphological analysis). All culture chambers were removed from the IAECs when back in the laboratory for analysis 10 days after the splashdown (January 24, 2022). The liquid in the housing of the culture chambers was withdrawn for measuring the medium-RNAlater or PFA/PBS exchanges to validate the fixation efficiency. The presence of cells on the glass slide was check by microscopy. The RNA was directly extracted and the samples for morphological analysis were kept at +4 °C in sterile PBS until use.

### RNA purification and next-generation sequencing

Total RNA was purified with High Pure RNA Isolation Kit, Roche #11828665001 from three distinct samples for each gravity condition. The quantity, quality and integrity of RNA were assessed using Agilent 2100 Bioanalyzer (Agilent). Purified total RNA was fragmented and, a double-stranded cDNA library was synthesized using the SMARTer Stranded RNA-Seq Kit (Takara #634836). The cDNA library was sequenced using an Illumina NextSeq 500 platform. Fastq files were mapped to Human reference genome (GRCh38 release 103) using the nf-core rnaseq pipeline (version 3.0, https://nf-co.re/rnaseq). The pipeline uses STAR (--aligner star_salmon) to map the raw fastq reads to the genome, to project the alignments onto the transcriptome and to perform the downstream BAM-level quantification with Salmon. We also added options in order to trim 5’ and 3’ T on forward and reverse reads and to trim based on quality after removing poly-G tails. For more information on software versions used along the pipeline, see nf-core documentation. Then, after QC report check, we used the per gene raw count matrix to perform differential expression analysis in R using DESeq2 package (https://bioconductor.org/packages/release/bioc/html/DESeq2.html). Genes were considered differentially expressed when the False Discovery Rate (FDR) was <0.01 using the B–H procedure.

### Pathways analysis

Gene sets were imported into the Ingenuity Pathways Analysis (IPA) software (QIAGEN Inc., https://www.qiagenbio-informatics.com/products/ingenuity-pathway-analysis) and subjected to functional annotations and regulatory network analysis using canonical signaling pathways, upstream regulator analysis and causal network analysis prediction algorithms.

### Cell staining and IF analysis

Cells were fixed onboard (4.75% paraformaldehyde in phosphate-buffered saline (PBS), pH 7.4, for 15 min) at the end of the experimental runs, washed twice with PBS and stored in PBS at +4 °C. Due to the relatively small sample size, areas of cell monolayers were delimited on each glass slide with PAP pen (Merck #Z672548) to realize all the immunostainings on the same sample simultaneously. Cell samples exposed to µg or 1 g in the ISS were treated in parallel with the same staining solutions, incubations times and washings.

The following primary antibodies were used: rabbit anti-KI67 (1:250, Abcam #ab16667), mouse anti-α-tubulin (1:1000, Sigma, #T6199), rabbit anti-acetyl-α-tubulin (1:500, Cell Signaling #5335P), mouse anti-LAMP2 (5 µg ml^−1^, DSHB #H4B4), rabbit anti-pFAK (1:500, Invitrogen #700255). The following secondary antibodies were used (1:500 dilution): Alexa Fluor 647 goat anti-rabbit (Thermo Fisher #A-21245), Alexa Fluor 488 goat anti-mouse (Thermo Fisher #A-21121), Alexa Fluor 488 goat anti-rabbit (Thermo Fisher #A-11008), Alexa Fluor 555 goat anti-rabbit (Thermo Fisher #A-21429), Alexa Fluor 555 goat anti-mouse (Thermo Fisher #A-21424).

Cells were permeabilized and blocked in 0.3% Triton X-100, BSA 5% in PBS for 1 h at RT. All the labeling were realized as follows: a 3 h-incubation at RT with the primary antibodies, 3 washes with BSA 1% in PBS, a 1-h-incubation with the secondary antibodies, 3 washes with BSA 1% in PBS, and nuclear staining with DAPI (0.25 μg ml^−1^, Life Technologies #D1306). Fibrillar actin staining was performed in 50 μg ml^−1^ of TRITC-conjugated phalloidin (Sigma-Aldrich, P1951) in BSA 1%. Antibodies and dyes were all diluted in BSA 1% in PBS.

Glass slides were then mounted with a coverslip using Fluoromount-G (Invitrogen, #00-4958-02) and imaged using Nikon TiS microscope with a Clara High Resolution CCD camera (Andor), halogen Fiber Illuminator Intensilight, Plan Apo 100× objective (Nikon) and controlled by NIS-Elements software (Nikon).

### Nuclear area, fluorescence intensity

The shape and size of DAPI-stained nuclei were analyzed on wide field images of samples stained with DAPI (~325 × 450 µm, Scan Large Image function) acquired with a ×100 magnification. The image reconstitution was realized by using a one-channel stitching with a 15% overlap (NIS-Elements software).

Nuclear area was measured after delineating each nucleus by using a binary mask and adding regions of interest (ROIs) by using NIS-Elements software. Each single nucleus was analyzed for its real surface in µm^2^ in the calibrated system. Intensity variations of DAPI in the nuclei were measured in the ROIs and expressed as the standard deviation of pixel intensities values. Intensity of KI67 staining in the nuclei was measured in the ROIs and expressed as the sum of pixel intensities.

### Statistical analysis

For each morphological measurement, an average of 130 nuclei were randomly selected (sampling is given below the figures in the results section). Data are presented as box plots and the used statistical procedures are indicated in the respective figures legends.

For IPA Core analyses, multiple pathways were tested for each analysis. Statistical tests were performed using Fisher’s exact test to assess which pathway was significantly associated with the genes in the dataset (total list of genes) after filtering on the base mean count, the log2 fold change and the adjusted *p* value as determined by the B–H method to control the false discovery rate (analysis-ready genes). The B–H *p* value was used to evaluate the statistical significance, under the null hypothesis, of the overlap between the analysis-ready genes and the genes reported to be part of defined pathways. A significance level of B–H *p* value <0.001 was set. The –log (B–H *p* value) and the *z*-score were used, as mentioned in the figure legends. An absolute *z*-score ≥2 is considered significant and relates to inhibition (negative *z*-score) or activation (positive *z*-score) of the pathways (http://pages.ingenuity.com/rs/ingenuity/images/0812%20downstream_effects_analysis_whitepaper.pdf).

In the Upstream Regulators analysis calculated by IPA, the purpose of the “overlap *p* value” (Fisher’s Exact Test, significance attributed to *p* values <0.001) is to identify regulators that could explain the observed gene expression changes. The overlap *p* value measures whether there is a statistically significant overlap between the dataset genes and the genes that are known to be regulated by a specific factor.

### Reporting summary

Further information on research design is available in the Nature Research Reporting Summary linked to this article.

### Supplementary information

Supplementary Notes

Supplementary Data 1

Supplementary Data 2

Supplementary Data 3

Supplementary Data 4

Supplementary Data 5

Supplementary Data 6

Supplementary Data 7

Supplementary Data 8

Reporting Summary

## Data Availability

All the necessary information needed for data interpretation is included in the manuscript. Any additional information might be provided upon request by contacting the corresponding author, Dr Alain Colige. Raw and processed data from next-generation sequencing have been archived in the NCBI GEO database: Accession GSE224805. Postflight dataset is already archived and available on HRE Data Archive: https://hreda.esac.esa.int/hreda/#/pages/investigation;investigationAcronym=Cytoskeleton_ISS.

## Code availability

Fastq files were mapped to Human reference genome (GRCh38 release 103) using the nf-core rnaseq pipeline at https://nf-co.re/rnaseq. R scripts using DESeq2 package are available at https://bioconductor.org/packages/release/bioc/html/DESeq2.html.
