# Supplementary material for: Microgravity triggers ferroptosis and accelerates senescence in the MG-63 cell model of osteoblastic cells
Source: NPJ Microgravity. 2023 Dec 16;9:91. doi: 10.1038/s41526-023-00339-3 (PMC10725437; doi:10.1038/s41526-023-00339-3)
Supplement: Supplementary file 1 — Supplementary Notes [file 41526_2023_339_MOESM1_ESM.pdf]

Supplementary Note 1

Transcriptomic comparison between the 1g on board and a ground experiment

Ground control (on Earth) experiment was carried out respecting the protocol used for the experiment on the ISS (hardware, software, cell culture and cryopreservation, schedules, temperature, medium exchanges, fixation and RNAseq methodology).

Comparisons have been made (IPA software) between the 1g-flight and a “ground” data, using similar cut-offs as those previously used for µg versus 1g comparisons (|log2 fold change| > 1, base mean count > 100, adjusted p-value < 0.001). Three canonical pathways were identified as significantly modified (|z-score| > 2 and -log (B-H p value) >2, Supplementary Figure 1 and Supplementary Data 1).

The most significant pathway predicted to be inhibited is the EIF2 signaling (z-score = -5.1; -log(B-H p-value = 26.4) with 53 altered genes out of 212 in the entire pathway. In our analysis, this pathway is mainly represented by ribosomal proteins L and S (RPLx and RPSx), which are involved in the assembly of either the small 40S or the large 60S ribosomal subunits. This indicates inhibition of the transcription machinery in 1g cultures in the ISS as compared to “ground” conditions. Furthermore, at another level of protein production, *AGO3*, which encodes for a protein involved in miRNA-mediated post-transcriptional gene silencing, was upregulated (fold change > 2), compared to ground control. The second pathway (Coronavirus pathogenesis) was identified essentially because of the regulation affecting RPS ribosomal proteins (as for the EIF2 pathway). Finally, the “Pulmonary Fibrosis Idiopathic Signaling” pathway was detected mainly based on the downregulations affecting genes coding for extracellular matrix proteins (collagens and fibronectin), growth factors and growth factor receptors (*ACVR2B*, *CCN4*, *FGF18*, *FGFR4*, *PDGFC*…). Remarkably, none of the pathways identified by comparing µg and 1g on board were significantly affected (|z-score|>2 and -log(B-H p-value >2). These additional comparisons of data from 1g cells on board and on Earth confirm the relevance of using onboard 1g culture as controls, as, otherwise, we would have concluded that the EIF2 pathway is altered by microgravity. On the other hand, we have no clues as to why the EIF2 pathway is repressed in space, although radiations may be the key.

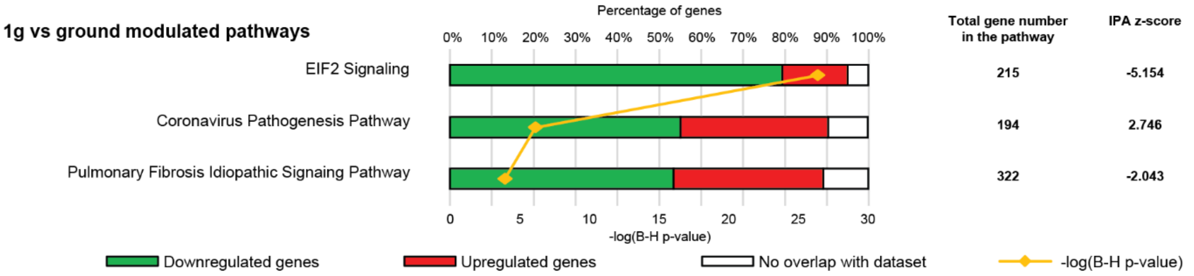

Supplementary Figure 1. Pathways associated to genes that are differentially expressed in 1g as compared to ground control. Three regulatory pathways were found to be modified in cells grown in the 1g centrifuge onboard as compared to cells grown on Earth. They are ranked according to their -log(B-H p-value), which is a corrected p-value based on the Benjamini-Hochberg method, a powerful tool for reducing the false discovery rate (yellow line and symbols). For each individual pathway, the percentage of genes that are significantly down-regulated, up-regulated and unregulated relative to our analysis thresholds is illustrated by a color code (green, red or white, respectively). The total number of genes in the pathway and the z-score calculated by IPA are also shown, with positive and negative z-score values indicating stimulation or inhibition respectively.

Supplementary Note 2

RT-qPCR validations of RNAseq analysis

cDNA from total RNA used in the RNAseq analysis was synthesized using a PrimeScript RT reagent Kit (Takara) according to the manufacturer's instructions. Resulting cDNA was subjected to quantitative real-time PCR using the SYBRgreen mix method (RT-SY2X-03+WOULR; Eurogentec). Real-time PCR experiments were run on a QuantStudio1 cycler (Thermofisher). Results were normalized using the geometrical mean of GAPDH, B2M and RPL4 transcripts. These three genes were similarly expressed in µg and 1g samples, as previously determined by RNAseq. Experiments were done in duplicate for the three biological replicates of each condition. Differences (n-fold) between samples were calculated using the ΔΔCt method. P-values were calculated using GraphPad Prism software (t-test). Primer sequences are listed in the Supplementary Table 2. A dilution curve of a reference cDNA was performed for each gene product to calculate the reaction efficiency. Correlation between RT-qPCR and RNAseq data was calculated by using the Spearman test for nonparametric values as implemented in the GraphPad Prism software.

As validation for RNAseq data, RT-qPCR assays were performed using the same RNA samples as in our transcriptomic analysis. Twelve gene products were selected according to their importance in the IPA analysis. A significant Spearman correlation was found between the RNAseq data and the RT-qPCR results (Supplementary Figure 2a and b). Altogether, these data demonstrate the reliability of the genome-wide transcriptomic analyses.

Supplementary Table 2. Oligonucleotide sequences of primers

**Gene symbol**	**Forward primer**	**Reverse primer**
GAPDH	5' CGCCCCACTTGATTTTGG 3'	5' ATGGAAATCCCATCACCATCT 3'
B2M	5' GAGTATGCCTGCCGTGTG 3'	5' AATCCAAATGCGGCATCT 3'
RPL4	5' ACCATGCGCCGGAACA 3'	5' CCACCCGGAGCTTGTGATT 3'
CCNB1	5' AGATCAGCACTCTACCACAGC 3'	5' TACACCTTTGCCACAGCCTT 3'
CENPE	5' GAGCTGCTTAGAGAAAAGGAAGA 3'	5' TGGGTCTTCAATGCTTTCCAATG 3'
CKAP2L	5' GGCGTTCGTCGAGGATTG 3'	5' CAGTTCATCAAGAGAAGCCACTACTAA 3'
E2F2	5' ATCCGCAAGAAGGCCAAGAA 3'	5' AAACATTCCCCTGCCTACCC 3'
FTH1	5' GTTCTTCGCCGAGAGTCGTC 3'	5' CGTCCAAGCACTGTTGAAGC 3'
IL1B	5' TCGCCAGTGAAATGATGGCT 3'	5' AGGTCCTGGAAGGAGCACTT 3'
IL6	5' CCCACCGGGAACGAAAGA 3'	5' CCGAAGGCGCTTGTGGAG 3'
LMNB1	5' GATGCAGCTCTTGCTACTGC 3'	5' GCTTCCAACTGGGCAATCTG 3'
NUPR1	5' GACTCCAGCCTGGATGAATCTG 3'	5' CTTCTCTCTTGGTGCGACCTTTC 3'
PTGS2	5' GTTCCACCCGCAGTACAGAA 3'	5' AGGGCTTCAGCATAAAGCGT 3'
YAP1	5' CGTCCAGCAAGATACTTTAATCCTCTAT 3'	5' ACTGTGAAAGAGGTCAGCAATACATT 3'
ACSL4	5' ATCATGTGGTGCTGGGACAG 3'	5' AAGAGGTGCTCCAACTCTGC 3'

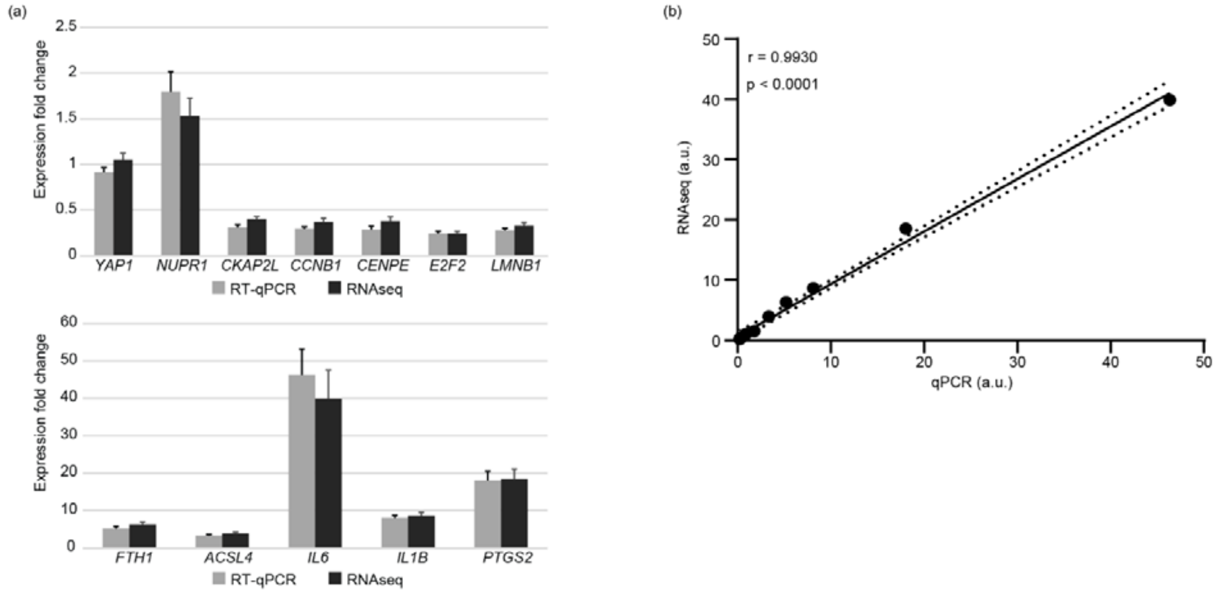

Supplementary Figure 2. RT-qPCR validations of RNAseq analysis. a Validation of RNAseq analysis results (comparing µg vs 1g) by RT-qPCR. RT-qPCR assays were performed in duplicate for the three biological replicates of each condition (µg and 1g). mRNA level quantifications by RT-qPCR were normalized by using data obtained for the three housekeeping gene transcripts (*GAPDH*, *B2M* and *RPL4*). Differences (n-fold) between samples (µg vs 1g) were calculated using the ΔΔCt method. P-values were calculated using GraphPad Prism software (t-test). Error bars represent SEM. **b** Correlation between the RNAseq data and RT-qPCR data. A linear regression was established between the changes in gene expression (in µg compared to 1g) as determined by RNAseq and RT-qPCR for 12 comparisons. $r = 0.99$ and $p < 0.0001$ using the Spearman test. Confidence interval set at 95%. Abbreviation a.u. = arbitrary units.

Supplementary Note 3

Comparison of the effects of microgravity and of the YAP inhibitor verteporfin

The YAP1 pathway was identified by IPA as a potential upstream regulator in microgravity. As a validation of the implication of YAP1 in the regulations observed in microgravity, we have tested the effect of verteporfin (SML0534, Sigma), a YAP1 inhibitor, which induces the sequestration of YAP in cytoplasm[1,2], and therefore interferes with its transcriptional activity. MG63 cells were treated with 10 µM verteporfin in DMSO for 32 hours and compared to control treated with DMSO alone. RNA was extracted as described in the method section and RT-qPCR were realized to assess the expression of 12 targets mRNA (including YAP1, NUPR1 and CKAP2L) and 3 housekeeping genes (see Supplementary Note 2). Comparisons (in fold changes) of the effects of verteporfin (versus DMSO) and of microgravity (versus 1g onboard) are provided in the Supplementary Figure 3.

Similar to what was observed for microgravity, verteporfin treatment causes a significant increase in the expression levels of genes linked to activated pathways (inflammation: *IL1B*, *IL6*, *PTGS2*; ferroptosis: *FTH1* and *ACSL4*), which further suggests the role of YAP1 in the microgravity-induced activation of these pathways. A decrease in the expression of *LMNB1* was also noticed as in microgravity. However, verteporfin did not affect the expression level of *CKAP2L*, *CENPE* and *E2F2*, which shows that mechanisms other than the YAP1 regulatory pathway could also be involved, probably mainly regarding genes for which the expression is reduced in microgravity.

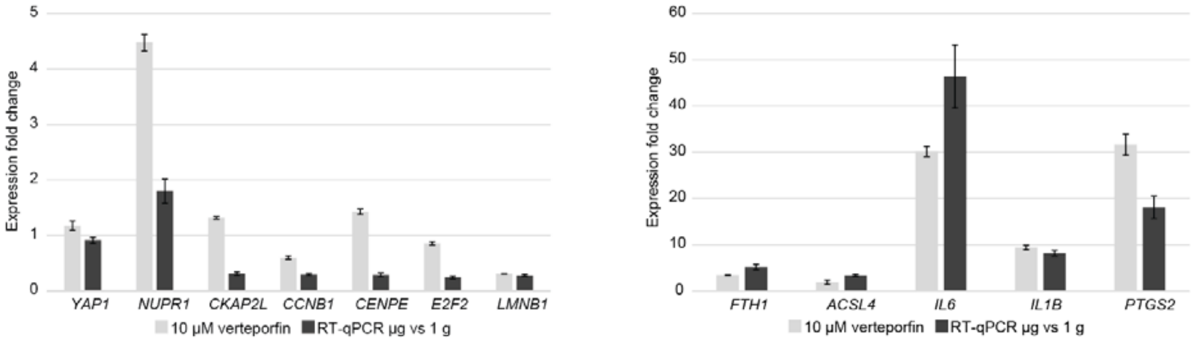

Supplementary Figure 3. Comparison of the effects of microgravity (vs control cultures at 1 g on board) and of the YAP inhibitor verteporfin (vs the DMSO control) on the expression levels (fold changes) of 12 mRNA targets. Error bars represent SEM.

1. Wang, C. *et al.* Verteporfin inhibits YAP function through up-regulating 14-3-3σ sequestering YAP in the cytoplasm. *Am. J. Cancer Res.* **6**, 27–37 (2016).

2. Giraud, J. *et al.* Verteporfin targeting YAP1/TAZ-TEAD transcriptional activity inhibits the tumorigenic properties of gastric cancer stem cells. *Int. J. Cancer* **146**, 2255–2267 (2020).
