# Supplementary material for: Microgravity triggers ferroptosis and accelerates senescence in the MG-63 cell model of osteoblastic cells
Source: NPJ Microgravity. 2023 Dec 16;9:91. doi: 10.1038/s41526-023-00339-3 (PMC10725437; doi:10.1038/s41526-023-00339-3)
Supplement: Supplementary file 10 — Reporting Summary [file 41526_2023_339_MOESM10_ESM.pdf]

nature portfolio

Corresponding author(s): Alain Colige

Last updated by author(s): Apr 13, 2023

Reporting Summary

Nature Portfolio wishes to improve the reproducibility of the work that we publish. This form provides structure for consistency and transparency in reporting. For further information on Nature Portfolio policies, see our Editorial Policies and the Editorial Policy Checklist.

Statistics

For all statistical analyses, confirm that the following items are present in the figure legend, table legend, main text, or Methods section.

n/a	Confirmed	
☐	☒	The exact sample size (*n*) for each experimental group/condition, given as a discrete number and unit of measurement
☐	☒	A statement on whether measurements were taken from distinct samples or whether the same sample was measured repeatedly
☐	☒	The statistical test(s) used AND whether they are one- or two-sided<br>*Only common tests should be described solely by name; describe more complex techniques in the Methods section.*
☒	☐	A description of all covariates tested
☐	☒	A description of any assumptions or corrections, such as tests of normality and adjustment for multiple comparisons
☐	☒	A full description of the statistical parameters including central tendency (e.g. means) or other basic estimates (e.g. regression coefficient) AND variation (e.g. standard deviation) or associated estimates of uncertainty (e.g. confidence intervals)
☐	☒	For null hypothesis testing, the test statistic (e.g. *F*, *t*, *r*) with confidence intervals, effect sizes, degrees of freedom and *P* value noted<br>*Give P values as exact values whenever suitable.*
☒	☐	For Bayesian analysis, information on the choice of priors and Markov chain Monte Carlo settings
☒	☐	For hierarchical and complex designs, identification of the appropriate level for tests and full reporting of outcomes
☒	☐	Estimates of effect sizes (e.g. Cohen's *d*, Pearson's *r*), indicating how they were calculated

Our web collection on statistics for biologists contains articles on many of the points above.

Software and code

Policy information about availability of computer code

Data collection	RNA quantity, quality and integrity : Agilent 2100 Bioanalyzer<br>cDNA sequencing: Illumina NextSeq 500<br>Image acquisition (microscopy): NIS-Elements software (Nikon)
Data analysis	Mapping to Human reference genome: the nf-core rnaseq pipeline (version 3.0, https://nf-co.re/rnaseq/3.0), STAR (--aligner star_salmon) to map the raw fastq reads<br>Differential expression analysis: R using DESeq2 package<br>QIAGEN Ingenuity Pathway Analysis (IPA)<br>Cellular morphology measurements: NIS-Elements software (Nikon), GraphPad Prism 9

For manuscripts utilizing custom algorithms or software that are central to the research but not yet described in published literature, software must be made available to editors and reviewers. We strongly encourage code deposition in a community repository (e.g. GitHub). See the Nature Portfolio guidelines for submitting code & software for further information.

Data

Policy information about availability of data

All manuscripts must include a data availability statement. This statement should provide the following information, where applicable:

- Accession codes, unique identifiers, or web links for publicly available datasets
- A description of any restrictions on data availability
- For clinical datasets or third party data, please ensure that the statement adheres to our policy

All data generated or analyzed during this study are included in the main text and its supplemental information files. The images used in this study are available from the corresponding author on reasonable request. RNAseq data have been archived in the NCBI GEO database: Accession GSE224805.

Human research participants

Policy information about studies involving human research participants and Sex and Gender in Research.

Reporting on sex and gender	*Use the terms sex (biological attribute) and gender (shaped by social and cultural circumstances) carefully in order to avoid confusing both terms. Indicate if findings apply to only one sex or gender; describe whether sex and gender were considered in study design whether sex and/or gender was determined based on self-reporting or assigned and methods used. Provide in the source data disaggregated sex and gender data where this information has been collected, and consent has been obtained for sharing of individual-level data; provide overall numbers in this Reporting Summary. Please state if this information has not been collected. Report sex- and gender-based analyses where performed, justify reasons for lack of sex- and gender-based analysis.*
Population characteristics	*Describe the covariate-relevant population characteristics of the human research participants (e.g. age, genotypic information, past and current diagnosis and treatment categories). If you filled out the behavioural & social sciences study design questions and have nothing to add here, write "See above."*
Recruitment	*Describe how participants were recruited. Outline any potential self-selection bias or other biases that may be present and how these are likely to impact results.*
Ethics oversight	*Identify the organization(s) that approved the study protocol.*

Note that full information on the approval of the study protocol must also be provided in the manuscript.

Field-specific reporting

Please select the one below that is the best fit for your research. If you are not sure, read the appropriate sections before making your selection.

☒ Life sciences ☐ Behavioural & social sciences ☐ Ecological, evolutionary & environmental sciences

For a reference copy of the document with all sections, see nature.com/documents/nr-reporting-summary-flat.pdf

Life sciences study design

All studies must disclose on these points even when the disclosure is negative.

Sample size	Sample size was based on ISS BioLab availability.
Data exclusions	No data was excluded from the analysis.
Replication	As the experiment was conducted on the ISS and based on ISS BioLab availability, one run was conducted for each condition. Three independent samples were collected for each run (RNA). For morphological analyses, one sample was divided to realize the different immunnostainings.
Randomization	Not relevant
Blinding	Not relevant as there was no group allocation.

Reporting for specific materials, systems and methods

We require information from authors about some types of materials, experimental systems and methods used in many studies. Here, indicate whether each material, system or method listed is relevant to your study. If you are not sure if a list item applies to your research, read the appropriate section before selecting a response.

Materials & experimental systems

n/a	Involved in the study
☐	☒ Antibodies
☐	☒ Eukaryotic cell lines
☒	☐ Palaeontology and archaeology
☒	☐ Animals and other organisms
☒	☐ Clinical data
☒	☐ Dual use research of concern

Methods

n/a	Involved in the study
☒	☐ ChIP-seq
☒	☐ Flow cytometry
☒	☐ MRI-based neuroimaging

Antibodies

Antibodies used

The following primary antibodies were used: rabbit anti-KI67 (Abcam #ab16667), mouse anti-α-tubulin (Sigma, #T6199), rabbit anti-acetyl-α-tubulin (Cell Signaling #5335P), mouse anti-LAMP2 (5DSHB #H4B4), rabbit anti-pFAK (Invitrogen #700255). The following secondary antibodies were used: Alexa Fluor 647 goat anti-rabbit (Thermofisher # A-21245), Alexa Fluor 488 goat anti-mouse (Thermofisher # A-21121), Alexa Fluor 488 goat anti-rabbit (Thermofisher # A-11008), Alexa Fluor 555 goat anti-rabbit (Thermofisher # A-21429), Alexa Fluor 555 goat anti-mouse (Thermofisher # A-21424).

Validation

rabbit anti-KI67 (Abcam #ab16667): knock-out validation (manufacturer's website)

mouse anti-a-tubulin (Sigma, #T6199): some references related to its use in immunocytochemistry: PMID: 31040275, PMID: 32737322, PMID24637461, PMID: 21159187, PMID: 19913571

rabbit anti-acetyl-a-tubulin (Cell Signaling #5335P): Cell Signaling validation steps include:
- Cell lines or tissues with known target expression levels are used to verify specificity.
- Appropriate cell lines and tissues are used to verify subcellular localization.
- Antibody performance is assessed on appropriate tissues.
- Cells are subjected to phosphatase treatment to verify phospho-specificity. Target specificity is also verified with the use of known knockout or null cell lines.
- Cells are subjected to siRNA treatment or over-expression of the target protein to verify target specificity.
- Activation state specification, target expression, and translocation are examined using ligands or inhibitors to modulate pathway activity.
- Requirement of threshold signal-to-noise ratio in antibody:isotype comparison and minimum fold-induction for phospho-specific antibodies ensures the greatest possible sensitivity.
- Fixation and permeabilization conditions are optimized; alternative protocols are recommended if necessary.
- Stringent testing ensures lot-to-lot consistency.

mouse anti-LAMP2 (DSHB #H4B4): some references related to its use in immunocytochemistry: PMID: 2912382, PMID: 18990578, PMID: 21161685, PMID: 11815981, PMID: 31302164, PMID: 3084996

rabbit anti-pFAK (Invitrogen #700255): validated by the manufacturer in A549 and in Hela cells and some references related to its use in immunocytochemistry: PMID: 31285581, PMID: 26056143, PMID: 35502265, PMID: 31471459, PMID: 32934005

Eukaryotic cell lines

Policy information about cell lines and Sex and Gender in Research

Cell line source(s)	MG63 cells (human osteosarcoma) from ATCC
Authentication	The cell line was authenticated by the information provided by ATCC.
Mycoplasma contamination	The MG63 cells were preventively treated with MP Biomedicals Mycoplasma Removal Agent according to the manufacturer's protocol. Then, they were tested and were negative for mycoplasma contamination.
Commonly misidentified lines (See ICLAC register)	Not relevant
